# Function of MYB8 in larch under PEG simulated drought stress

**DOI:** 10.1038/s41598-024-61510-8

**Published:** 2024-05-17

**Authors:** Qingrong Zhao, Huanhuan Xiong, Hongying Yu, Chen Wang, Sufang Zhang, Junfei Hao, Junhui Wang, Hanguo Zhang, Lei Zhang

**Affiliations:** 1 State Key Laboratory of Tree Genetics and Breeding (Northeast Forestry University), Harbin, China; 2 Forestry Research Institute in Heilongjiang Province, Harbin, China; 3 State Administration of Forestry and Grassland, Harbin Research Institute of Forestry Machinery, Harbin, China; 4 College of Forestry and Landscape Architecture, South China Agricultural University, Guangzhou, China; 5 State Key Laboratory of Tree Genetics and Breeding (Chinese Academy of Forestry), Beijing, China

**Keywords:** *Larix spp.*, MYB, PEG stress, Transient genetic transformation, Drought, Gene expression analysis, Genomic analysis

## Abstract

Larch, a prominent afforestation, and timber species in northeastern China, faces growth limitations due to drought. To further investigate the mechanism of larch’s drought resistance, we conducted full-length sequencing on embryonic callus subjected to PEG-simulated drought stress. The sequencing results revealed that the differentially expressed genes (DEGs) primarily played roles in cellular activities and cell components, with molecular functions such as binding, catalytic activity, and transport activity. Furthermore, the DEGs showed significant enrichment in pathways related to protein processing, starch and sucrose metabolism, benzose-glucuronic acid interconversion, phenylpropyl biology, flavonoid biosynthesis, as well as nitrogen metabolism and alanine, aspartic acid, and glutamic acid metabolism. Consequently, the transcription factor T_transcript_77027, which is involved in multiple pathways, was selected as a candidate gene for subsequent drought stress resistance tests. Under PEG-simulated drought stress, the LoMYB8 gene was induced and showed significantly upregulated expression compared to the control. Physiological indices demonstrated an improved drought resistance in the transgenic plants. After 48 h of PEG stress, the transcriptome sequencing results of the transiently transformed LoMYB8 plants and control plants exhibited that genes were significantly enriched in biological process, cellular component and molecular function. Function analyses indicated for the enrichment of multiple KEGG pathways, including energy synthesis, metabolic pathways, antioxidant pathways, and other relevant processes. The pathways annotated by the differential metabolites mainly encompassed signal transduction, carbohydrate metabolism, amino acid metabolism, and flavonoid metabolism.

## Introduction

The *Larix *spp., a deciduous tree belonging to the pine family, is renowned for its impressive height, exceptional cold tolerance, and rapid growth rate. During its early stages of development, the larch exhibits vigorous growth, making it highly suitable for the afforestation^1^. Moreover, it serves as a primary source of timber and plays afforestation efforts in northeast China^2^. Moreover, larch species possess large genomes and intricate genetic backgrounds. Regrettably, the absence of transcriptome sequencing studies and publications specifically for larch has hindered the exploration and application of drought resistance genes. Enhancing productivity, wood quality, and resilience to biological and abiotic stresses through tree genetic engineering has been a primary objective in larch forestry biotechnology community for decades. Despite numerous challenges, significant progress has been made in tree biotechnology in recent years^3^.

Drought is undeniably one of the most significant environmental issues faced globally. In arid regions, plants undergo a multitude of physiological and development changes throughout their growth stages. Unraveling the mechanisms that enable plants to maintain productivity in adverse conditions, particularly drought, and harnessing these mechanisms to enhance plant adaptability to environmental fluctuations remain paramount challenges in the realm of plant research^4,4^. When confronted with drought stress, plants employ various protective strategies to ensure their survival, including modifications in root and leaf morphology^6^, adjustments in metabolite profiles^7^, and regulation of drought resistance gene expression^8^. Drought conditions can significantly impact plant water potential and increase the vulnerability of xylem to weathering. Research has indicated that larch, compared to other coniferous species, exhibits greater sensitivity to soil moisture and experiences slower growth under drought conditions. Additionally, the early stages of plant growth are particularly critical for larch, as water loss during this period can result in stunted growth or even mortality. While significant attention has been dedicated to exploring the response to drought stress in broad-leaved tree species and crops such as birch^9^, poplar^10^, soybean^11^, maize^12^, the studies on larch are notably scarce. Therefore, a comprehensive investigation into the drought resistance mechanisms of larch. In this study, transcriptome sequencing was performed with PEG treatment of the used plant material, and a MYB family gene with drought-resistant functions, T_transcript_77027, was identified. It was subsequently named LoMYB8.

The MYB family is one of the largest transcription factor families in plants and has the most members with the most diversified functions^13^. These transcription factors contain a specific MYB domain that can induce the expression of downstream genes. Many transcription factors containing an MYB domain in animals and plants have since been identified and isolated, resulting in the classification of these transcription factors into a new gene family. A number of studies have shown that MYB transcription factors are involved in the response of plants to drought stress, offering an avenue for the improvement of drought resistance in plants. Liao et al. identified 156 GmMYB genes in soybean, 43 of which were involved in the response to drought stress under abscisic acid (ABA) induction^14^. In *Arabidopsis*, *AtMYB44*/*AtMYBR1*, *AtMYB60*, *AtMYB13*, *AtMYB15*, and *AtMYB96* control the degree of stomatal opening by regulating the accumulation of ABA to enhance the tolerance of plants to drought^15^. MYB transcription factors are also related to drought-stress responses in poplar^16^, apple^17^, and Jatropha curcas^18^. Several studies have shown that most of the MYB transcription factors involved in plant drought-stress responses are R2R3-MYB TFs with two R structures, which is the type of MYB that has the most members in plants^19,19,19,19,19^. MYB transcription factors play a role in the drought-resistance response of plants via various mechanisms that are mostly related to ABA^22,22,22^ and light signalling^23^.

## Materials and methods

### cDNA library construction and transcriptome sequencing

The embryogenic callus of Hybrid larch in the laboratory using immature zygote embryos. Careful selection good development and stable growth for further experiments. Subsequently, these callus samples were treated with 5% PEG6000 for different durations: 0 h (CK), 12 h (T1), 24 h (T2), and 48 h (T3). At each time point, three repetitions were performed to ensure sample reliability. After rapid freezing with liquid nitrogen, the samples were stored at − 80 °C and then transported to Beijing for sequencing analysis. Prior to constructing the cDNA library, all samples underwent quality testing. Once the quality assessment was completed, construction of both Illumina and PacBio cDNA libraries took place using magnetic bead enrichment method followed by computer-based sequencing. The Illumina HiSeq 2500 system from Illumina in San Diego, CA, USA was used for sequencing the cDNA library alongside full-length transcriptome sequencing performed by PacBio instrument.

### Screening, annotation and analysis of DEGs

In Differentially Expressed Transcripts analysis, to obtain comprehensive annotation information for DEGs, they can be compared against various databases such as NR^59^, Swissprot^60^, GO^61^, COG^62^, KOG^63^, Pfam^64^, and KEGG^65^.

### Quantification and verification of gene expression levels

To validate the accuracy of the RNA-seq analysis, RT-qPCR analysis was performed on 17 DEGs selected from the predicted DEGs in response to drought stress. RNA was extracted from embryonic callus using the Universal Plant Total RNA Extraction Kit (BIOTEKE, Beijing, China), and cDNA was synthesized using the PrimeScriptTM RT reagent Kit with gDNA Eraser (Perfect Real Time) (TaKaRa Biotech, Dalian, China). RT-qPCR primers were designed using Primer5 software (Table 1). Quantitative fluorescence analysis was performed using TB Green® Premix Ex Taq^TM^II (Tli RNaseH Plus) kit (TaKaRa Biotech, Dalian, China). Each gene was analyzed with three replicates using an ABI7500 fluorescence quantitative PCR instrument. Data analysis was conducted using Microsoft Excel 2016, and the results were analyzed using the 2-ΔΔCt method, with α-tubulin serving as the reference gene for normalization (NCBI accession number MF278617.1).

**Table 1:** Primers used in real-time RT-PCR.

Gene name	Forward primers(5′-3′)	Reverse primers(5′-3′)	TM (°C)	GC(%)	Amplicon length(bp)
T_120312	CTCTGAAGTGTCCGCTCTAT	AACCCTTCTTGGTAGTTGTAAT	52	50/36.4	113
T_95607	GCTTCCTCCACATCGTTTA	TCATTCACAGCGACTTCTT	55	50	93
T_3739	ATGAGACCACTACATTGGACGAT	GCAAGGGAGTCTGCTGTGA	59	39.1/63.2	95
T_58622	GGTCCCTGTAGTGGTTCGC	CAGCAGCATCATCCAGATT	56	45/40	91
T_92243	AGGGAAACGCTGCTCAAAC	GAGGACCTGCTGCCTGGAT	57	52.6	103
T_13694	ACCAAGATGCGATTGATGC	CTGTTCGTTTGTCAGTGGG	57	52.6	97
T_11943	ATTTACTAGGGCGTTCTGTC	TGCTGGTTACCGTTATTACTC	56	50	120
T_19684	TGCAGCATTTCAAGCTCAC	ACGATTCGCCCAATACACT	58	40	94
T_102009	ACGAATAGAAGCAGCAGGAG	TGATGTTGGACCCAGTTTG	54	50/42.9	115
T_13048	ATGGAGGAAGCCGTAGTGG	ATCATCATAAGAACGTGTCCCAC	55	57.9/52.6	116
T_50849	TGAACACCCTGATCGACTT	TCCTTGTACGGGCATACTT	55	40.9/45.5	105
T_24588	CTATCTATGTTGAATGCGGTGGA	GGGATCTGGCTGCAATGGT	56	50/45	103
T_37604	TCCAAATCAACAGGCAATG	AAGCCACAAATGGTGAGTAAG	57	40/40.9	110
T_90525	CTTTCAGTGGTTGGAGGAG	ATTCTTGGGAGGGATTTGT	56	55.6	95
T_114983	GGCTCGGAGAAGAGGACAT	CGAACAACTATTGAGGGATTTA	57	55.4	119
T_118082	ATGAATGCTCTGGCTGCAACA	TGTTGCAGCCAGAGCATTCAT	55	50	107
T_28317	TCAGAAAGGACCTACTACCC	ATTTGTTGTGCCCTGTTTA	55	45/45	114

### Transient genetic transformation of larch seedlings

Thirty to Forty Five-day-old seedlings of larch lacking a fully expanded needle leaf were selected for transient genetic transformation. The seedlings were soaked in hypertonic solution for 10 min and then transferred to a container with a liquid suspension of bacteria (laboratory Agrobacterium strains, GV3101), and the air pressure in the container was pumped down for 10 min. The container was then placed on a shaker at a constant temperature of 26 °C and 120 rpm for 4 h. The infected seedlings were rinsed three times with sterilized water, and the water remaining on the seedlings was removed using sterilized filter paper. The seedlings were then cultured in sterilized soil mix and covered with a plastic membrane to retain moisture. After 48 h, the seedlings were removed from the soil mix and rinsed with sterilized water, with the remaining water on the seedlings removed using sterilized filter paper.

The 1 mol/L mannitol hypertonic solution was prepared as follows: 182.17 g mannitol powder was weighed and completely dissolved in 1 L deionized water with stirring, and then the solution was placed at room temperature for immediate use. The ingredients of the infection solution for larch transformation included sucrose 3%, KT 1.5 mg/L, 2,4-D 5.0 mg/L, CaCl2 10 mmol/L, MgCl2 10 mmol/L, sucrose 3%, coniferyl alcohol 100 μmol/L, mannitol 400 mmol/L, DTT 0.2 g, Tween 0.05% (v/v), and MES 10 mmol/L at pH 5.6. 

### PEG treatment

PEG solution (20%, w/v) was prepared and used to waterize the transiently transformed plants that were cultured in a soil mix. The treatment started after the transiently transformed plants were cultivated in soil mix for 12 h, and the samples taken at this time were labelled MYB8-T0. Following PEG treatment, samples were taken at 24 h and 48 h and were labelled MYB8-T24 and MYB8-T48, respectively. The plants transformed with an empty vector were used as the control, and the samples were taken in the same way and labelled CK-T0, CK-T24, and CK-T48, respectively.

### RNA extraction and RT-qPCR

Genomic RNA was extracted from fresh tissue by the CTAB method described by Chang et al.^24^, total RNA was treated with DNase I (TaKaRa Bio, Shiga, Japan) to remove genomic DNA, and then the concentration was measured using a NanoDrop 2000 (Thermo). RT-qPCR method is the same as above.

### Biochemical marker determination

All experimental variants were taken for biochemical analysis. Each sample had three biological replicates, consisting of three seedlings. The soluble sugar content was determined by using the principle of anthrone colorimetry^25^, the soluble protein content was determined by using Coomassie Brilliant Blue G-250^26^, and the peroxidase (POD) activity (G0107F, Grace Biotechnology Co., Ltd., Suzhou), malondialdehyde (MDA) content (G0109F, Grace Biotechnology Co., Ltd., Suzhou) and superoxide dismutase (SOD) activity were detected using a kit(G0103F, Grace Biotechnology Co., Ltd. Suzhou).

### Differential gene expression and metabolite analysis

The analysis of differential gene expression is described above. Sequencing libraries were generated using the NEBNext®Ultra™ RNA Library Prep Kit for Illumina® (NEB, USA) following the manufacturer’s recommendations, and index codes were added to attribute sequences to each sample (CK-T48 and OE-T48).

Samples were thawed on ice at 4 °C. Subsequently, 100 μL of each sample was transferred to an EP tube and extracted using 300 μL of methanol. Following this, 20 μL of internal standard substances were added. The samples were vortexed for 30 s, sonicated for 10 min (while incubating in ice water), and then incubated for 1 h at − 20 °C to precipitate proteins. Afterward, the samples were centrifuged at 13,000 rpm for 15 min at 4 °C. The supernatant (200 μL) was carefully transferred to a fresh 2 mL LC/MS glass vial. Additionally, 20 μL of each sample was pooled to create quality control (QC) samples. A 200 μL aliquot of the supernatant was designated for UHPLC-QTOF-MS analysis.The specific analysis method has been referred to tian's method^27^.LC–MS/MS analyses were conducted utilizing an UHPLC system (model 1290, Agilent Technologies). The MS raw data (.d) files were converted to the mzXML format using ProteoWizard and processed using the R package XCMS (https://bioconductor.org/packages/release/bioc/html/xcms.html).

### Ethical approval

Research and field studies on plants (either cultivated or wild), including the collection of plant material, was carried out in accordance with relevant institutional, national, and international guidelines and legislation

## Results

### Statistical analysis of sequence data

On the Illumina HiSeq platform, a total of 20.3 Gb of high-quality reads were generated. The GC content of the 12 samples ranged from 44.53 to 44.91%, while the Q30 content varied between 94.07% and 96.14% (Table 2). For full-length transcriptome sequencing using the PacBio platform, we obtained a total of 786,492 circular consensus sequence (CCS) reads with an average length of approximately 3,310 bp. Detailed library information including CCS read count, base count, and average length can be found in Table 2 in the appendix section. Among these CCS reads, there were a total of 393,246 full-length (FL) reads and 340,254 non-chimeric full-length (FLNC) reads.

**Table 2:** Statistics of Illumina HiSeq clean reads**.**

Samples	ReadSum	BaseSum	GC (%)	CycleQ20 (%)	Q20 (%)	Q30 (%)
CK-1	24,907,448	7,425,001,256	44.75	100	98.49	95.16
CK-2	26,564,911	7,940,793,372	44.77	100	98.21	94.51
CK-3	24,444,550	7,304,446,818	44.64	100	98.28	94.67
T1-1	30,880,265	9,222,115,228	44.74	100	98.2	94.48
T1-2	31,819,158	9,496,352,584	44.78	100	98.12	94.29
T1-3	28,009,783	8,348,200,782	44.84	100	98	94.07
T2-1	27,569,258	8,235,269,454	44.74	100	98.82	95.82
T2-2	23,405,579	7,012,171,264	44.91	100	98.91	96.14
T2-3	22,204,401	6,646,909,002	44.53	100	98.77	95.84
T3-1	23,583,276	7,036,645,640	44.84	100	98.37	94.86
T3-2	25,788,191	7,687,972,930	44.75	100	98.32	94.78
T3-3	25,650,706	7,649,303,868	44.89	100	98.41	94.98

### Screening, functional annotation and enrichment of DEGs

**Figure 1:**
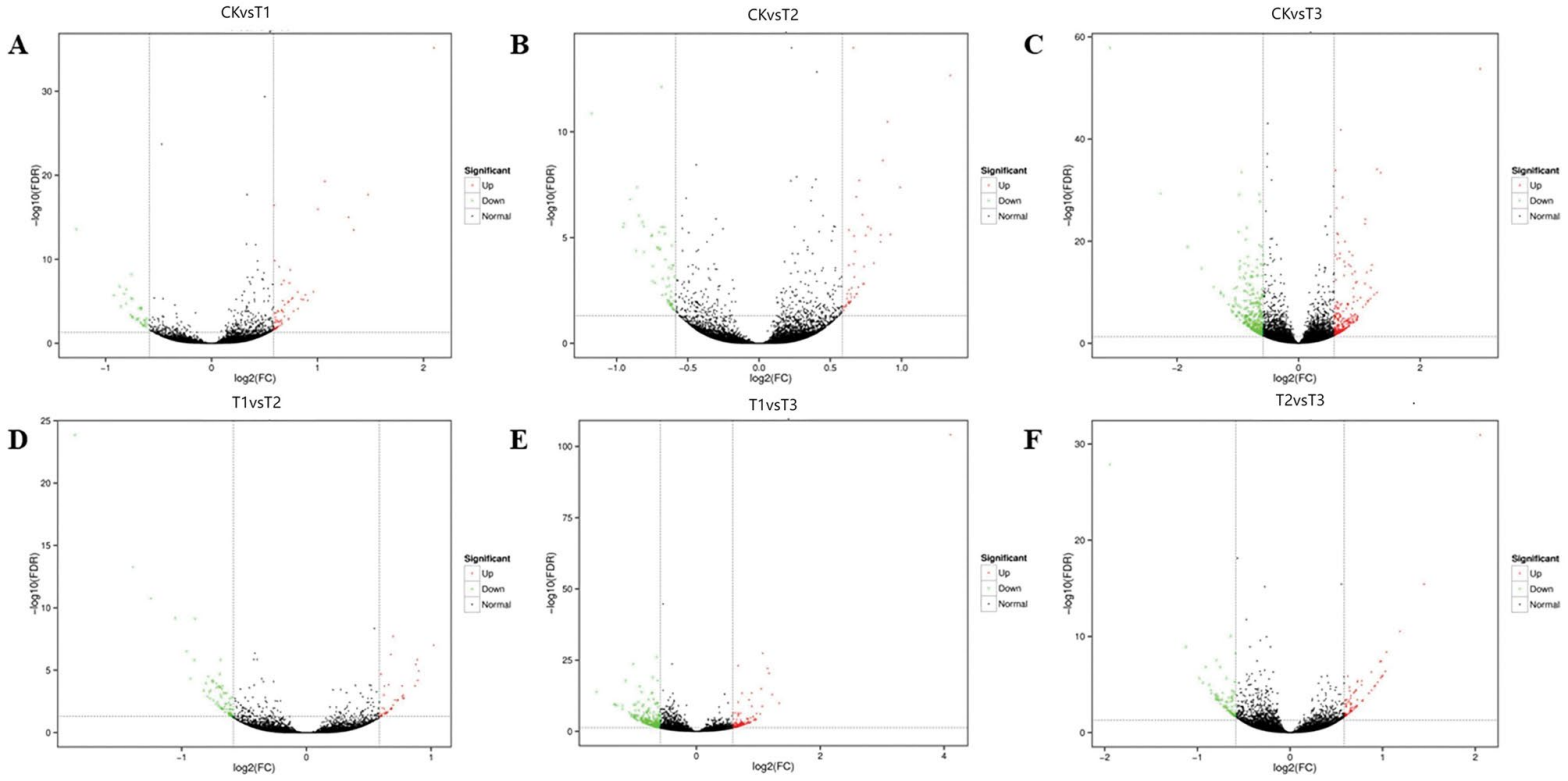
The DEG’s Volcano Plot. *Note*: (**A**) is CK versus T1, (**B**) is CK versus T2, (**C**) is CK versus T3, (**D**) is T1 versus T2, (**E**) is T1 versus T3, (**F**) is T2 versus T3.

The transcriptome of embryogenic callus under drought stress was sequenced to identify DEGs. According to the screening criteria (Fold Change ≥ 1.50 and FDR < 0.05), a total of 1,654 DEGs were identified (Fig. 1). In calli subjected to 3d-long stress, the highest number of both upregulated and downregulated DEGs was detecred. These findings suggest that the embryonic callus initiated the regulation of DEG expression, thus responding to drought stress, following a 48-h simulation of drought stress using PEG.

Functional annotations of DEGs are made using the database (Table 3). Out of a total of 1,580 DEGs, the majority (99.8%) were successfully annotated using the NR database, indicating high accuracy and coverage of the annotation process.

**Table 3:** Annotated DEGs’ datasets in public databases**.**

Samples	Annotated	COG	GO	KEGG	KOG	Pfam	Swissprot	eggNOG	NR
CK versus T1	99	56	73	53	74	91	82	97	99
CK versus T2	100	52	75	58	78	96	85	100	100
CK versus T3	676	351	486	320	411	593	527	658	674
T1 versus T2	109	56	88	60	77	101	83	106	108
T1 versus T3	445	246	340	236	305	404	353	432	445
T2 versus T3	151	70	100	82	109	132	113	146	151

**Figure 2:**
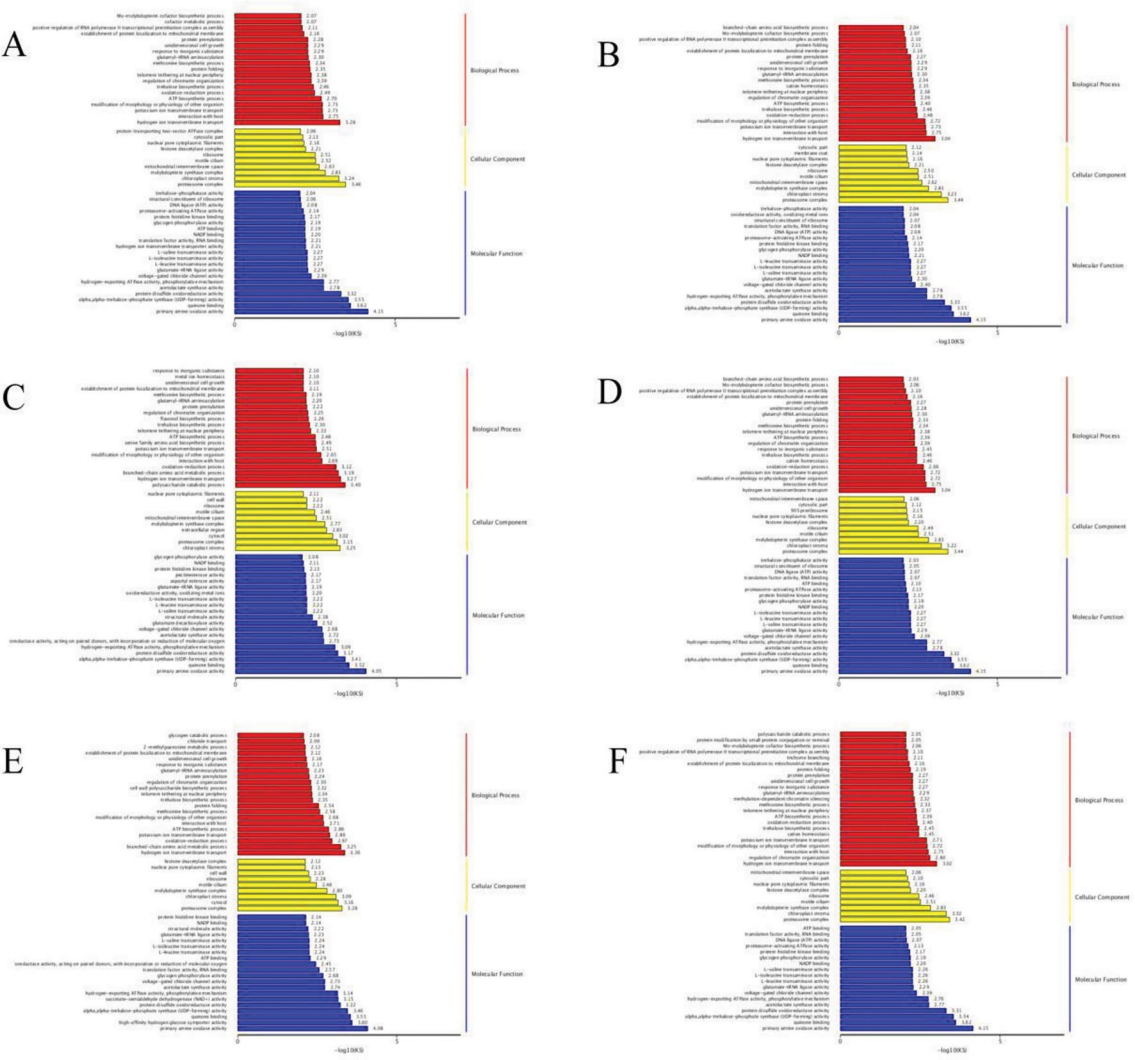
Statistics of the classification of the GO annotations for the DEG**.** *Note*: (**A**) is CK versus T1, (**B**) is CK versus T2, (**C**) is CK versus T3, (**D**) is T1 versus T2, (**E**) is T1 versus T3, (**F**) is T2 versus T3**.**

Figure 2 illustrates that the DEGs from the six comparisons in Table 3 predominantly exhibit involvement in distinct tissue processes within metabolic, cellular and biological processes. Specifically, these DEGs are associated with cellular components such as cells, cell parts, organelles, and membranes, while performing molecular functions such as binding, catalytic activity, and transporter activity. Based on these findings, it is reasonable to speculate that in larch trees, the reception of drought signals from the external environment triggers a series of signal transduction cascades. These cascades subsequently activate transcription factors (TFs) response of the relevant function, promoting the synthesis of metabolites that enable the tree to respond and cope with drought conditions.

**Figure 3:**
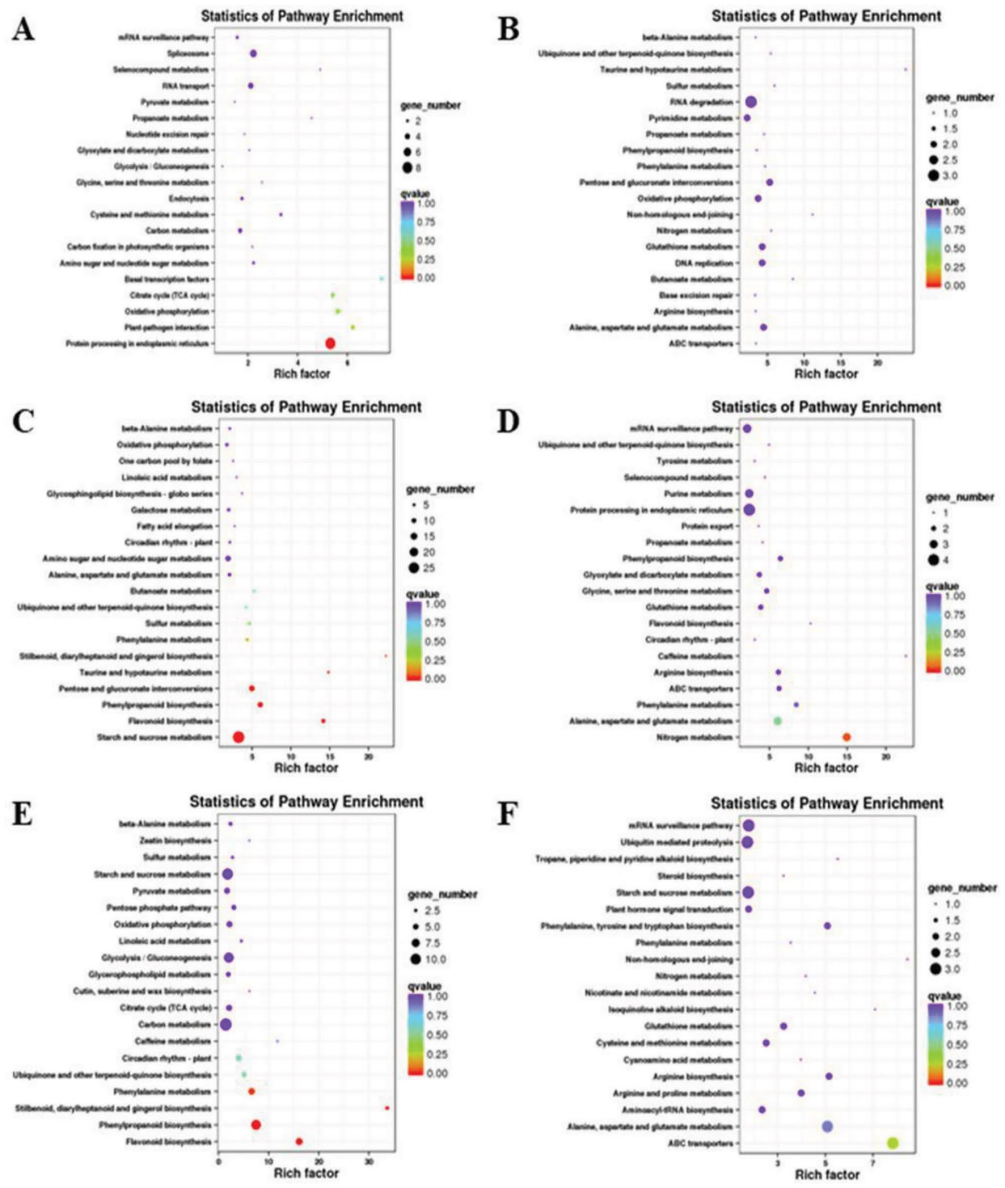
Statistics of KEGG enrichment for DEGs. *Note*: (**A**) is CK versus T1, (**B**) is CK versus T2, (**C**) is CK versus T3, (**D**) is T1 versus T2, (**E**) is T1 versus T3, (**F**) is T2 versus T3.

In organisms, different gene products collaborate with one another to carry out biological functions. Pathway enrichment analysis of DEGs aids in determining whether these genes are are over-represented in specific pathways. In the 6 control groups, KEGG enrichment analysis was performed on the DEGs, resulting in Fig. 3, which displays the top 20 pathways with the lowest significant q-values. In the CKvsT1 comparison, DEGs were notably enriched in protein processing pathways, as well as several others. In CKvsT2, the enrichment significance of DEGs was comparatively low, with a higher enrichment observed in the RNA degradation pathway. On the other hand, in CKvsT3, DEGs were significantly enriched in pathways related to starch and sucrose metabolism, phentose-glucuronate interconversion, and phenyl-C biosynthesis. Regarding the T1vsT2 comparison, DEGs exhibited significant enrichment in nitrogen metabolism, as well as the metabolism of alanine, aspartic acid, and glutamic acid. For T1vsT3, DEGs showed significant enrichment in phenyl-C biosynthesis, phenylalanine metabolism, and flavonoid biosynthesis pathways. Lastly, in the T2vsT3 comparison, DEGs were significantly enriched in ABC transporters. These pathways provide valuable insights into the metabolic information of embryogenic callus in hybrid larch under drought stress. Furthermore, they contribute to a better understanding of the potential regulatory mechanism associated with drought resistance. Based on the above analysis, some of the DEGs which have been significantly enriched in GO analysis and also in the KEGG pathway have been selected, and the gene T_transcript_77027 has been selected from them for verification.

### Quantification and verification of DEGs expression

**Figure 4:**
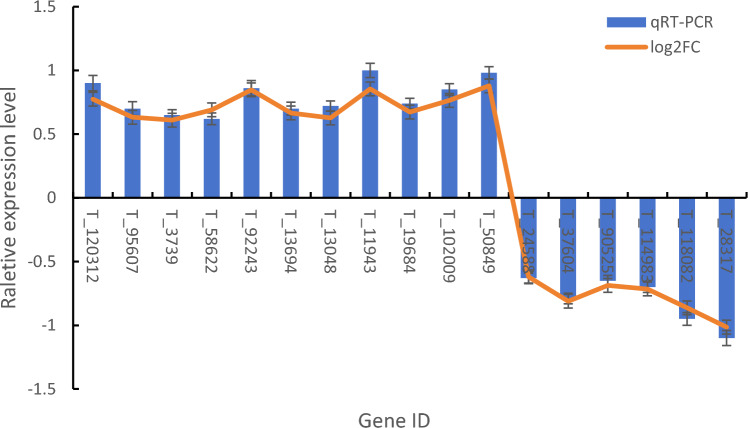
The relative expression levels of 20 DEGs between RNA-seq and RT-qPCR.

To validate the accuracy of RNA-seq analysis of hybrid larch under drought stress, we selected 17 DEGs from genes form RNA-seq. Figure 4 demonstrates a high level of agreement between the RT-qPCR results and the RNA-seq data for these 17 DEGs. While there may be some variations in the expression levels, the overall expression patterns remained consistent with RNA-seq results, indicating the reliability and authenticity of the RNA-seq findings.

The results showed that more MYB genes were identified in transcriptome sequencing, and MYB genes were mainly enriched in the cellular transport active molecular pathway, while in the KEGG pathway, MYB genes were mainly enriched in sucrose metabolism, phenyl-propyl biosynthesis, glutamate metabolism, and flavonoid biosynthesis pathway. Meanwhile, in previous studies ^28^, it was found that MYB gene was also expressed in secondary xylem of stems and roots. At the same time, the MYB gene family plays a role in the development process and defense response of plants, so the MYB8 gene was screened in the transcriptome, and subsequent tests were conducted to verify whether the gene has drought resistance.

### Gene expression of transiently transformed seedlings

**Figure 5:**
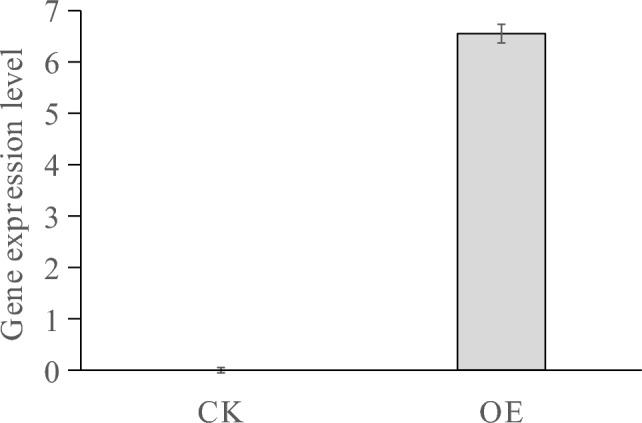
Gene expression in the LoMYB8 overexpression plants obtained by instantaneous transformation.

The expression level of the LoMYB8 gene in transiently transformed larch seedlings was 6.55 times higher than that in the control plants, which confirmed that the transformation system for larch effectively resulted in the overexpression of the LoMYB8 gene (Fig. 5).

**Figure 6:**
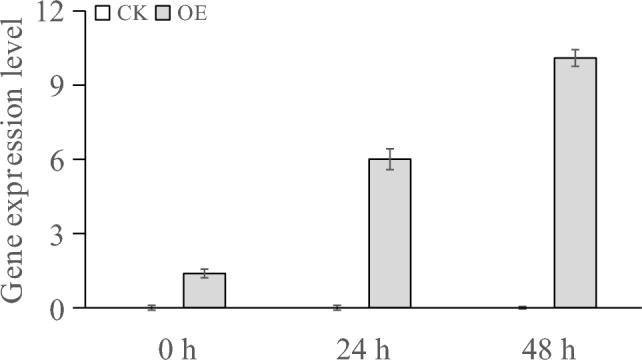
Gene expression in transiently transformed LoMYB8 overexpression larch plants under PEG stress.

The expression level of the *LoMYB8* gene in the transiently transformed plants under PEG-simulated drought stress was significantly higher than that in the unstressed transiently transformed plants at the same time points (Fig. 6). The expression level of the *LoMYB8* gene increased significantly in the transiently transformed plants treated with PEG for 24 h and reached the same expression level as that in the untreated transiently transformed plants at 48 h. Following treatment with PEG for 48 h, the expression level of the *LoMYB8* gene appeared continuously upregulated.

### Biochemical indicators in transiently transformed seedlings under PEG stress

**Figure 7:**
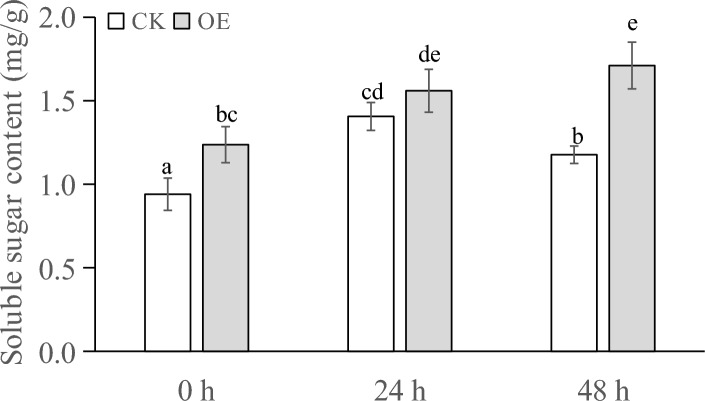
Soluble sugar content (mg/g) of transgenic plants under PEG stress at different times.

The soluble sugar contents of the plants that had undergone different treatments are shown in Fig. 7. At 0 h, the soluble sugar content of the transiently transformed plants was slightly higher than that of the control plants. After 24 h of PEG treatment, the soluble sugar content of both the treated plants and control plants increased, and the soluble sugar content of the transiently transformed plants was approximately 1.14 times higher than that of the control plants, but the difference was nonsignificant. After 48 h of PEG treatment, the soluble sugar content of the transiently transformed plants was 1.5 times higher than that of the control plants, and the difference was significant. With the increase in treatment duration, the soluble sugar content of the control plants first increased and then decreased, while the soluble sugar content of the transiently transformed plants continued to increase.

**Figure 8:**
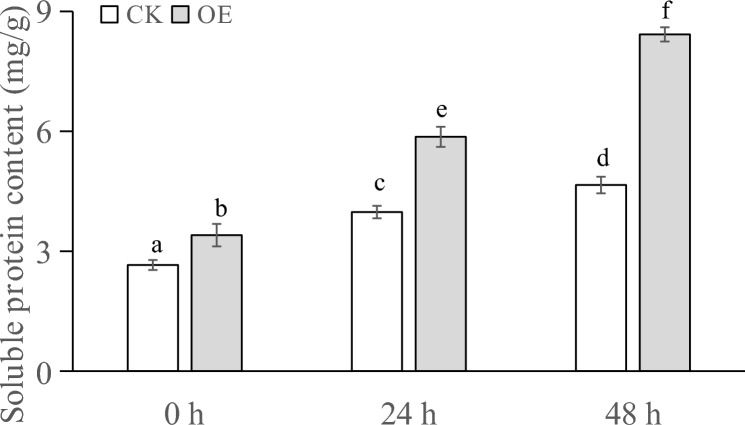
Soluble protein content (mg/g) of transgenic plants under PEG stress at different times.

The soluble protein contents in the plants that had undergone different PEG treatments are indicated in Fig. 8. At T0, the soluble protein content in the transiently transformed plants was slightly higher than that in the control plants. After 24 h of stress treatment, the soluble protein content in both the control plants and transiently transformed plants increased. The soluble protein content in the transiently transformed plants was 1.5 times higher than that at 0 h of treatment, and the difference was significant. After 48 h of stress, the transiently transformed plants showed a significantly higher soluble protein content than those that had been stressed for 24 h, and the soluble protein content in the transiently transformed plants was significantly higher than that in the control plants.

**Figure 9:**
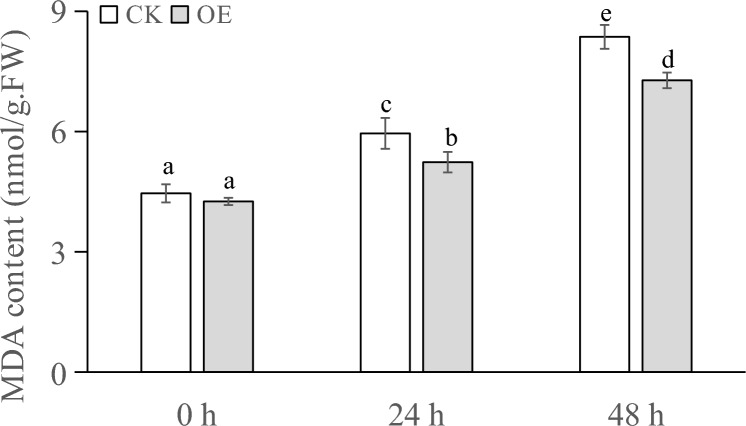
MDA content (nmol/g·FW) of transgenic plants under PEG stress at different times.

The MDA contents of the plants sampled from the different PEG treatments are shown in Fig. 9. At 0 h, the difference in MDA content between the transiently transformed plants and control plants was nonsignificant. At 24 h, both the transiently transformed plants and control plants showed an increased MDA content, although the MDA content in the transiently transformed plants was lower than that in the control plants. At 48 h, the MDA content in the transiently transformed plants was significantly lower than that in the control plants.

**Figure 10:**
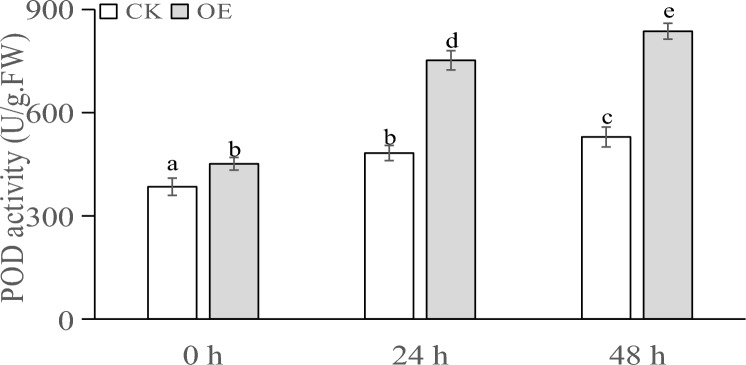
POD activity (U/g) of transgenic plants under PEG stress at different times.

The POD activity in the plants under different treatments is shown in Fig. 10. At 0 h, the POD activity in all the plants was basically the same. At 24 h, the POD activity in both the transiently transformed and control plants increased, and the POD activity in the transiently transformed plants was significantly higher than that in the control plants. At 48 h, the POD activity in both the transiently transformed and control plants increased, but the increase in POD activity in the control plants was not obvious. The increase in POD activity in the transiently transformed plants was higher than that in the control plants.

**Figure 11:**
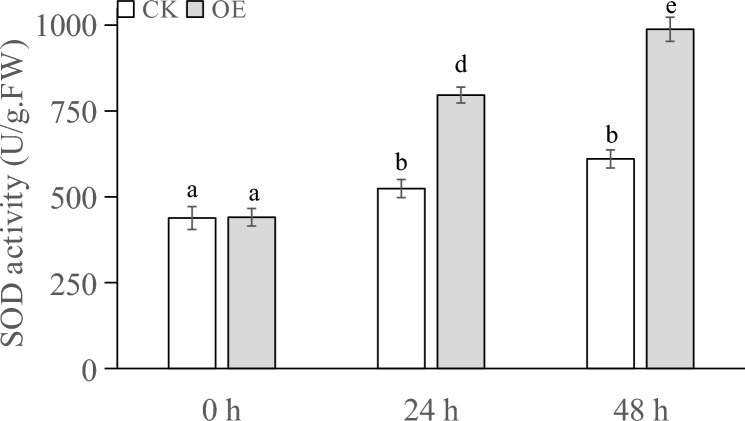
SOD activity (U/g) of transgenic plants under PEG stress at different timepoints.

The SOD activity in the plants under different treatments is shown in Fig. 11. At 0 h, the activity of SOD enzymes in all the plants was basically the same. At 24 h, the SOD activity in the control plants slightly increased, and the increase in SOD activity in the transiently transformed plants was higher than that in the control plants. At 48 h, the increase in SOD enzyme activity in the transiently transformed plants was higher than that in the control plants.

When plants are subjected to drought stress, they generally reduce the cellular osmotic potential to prevent excessive cellular water loss by reducing the intracellular water content, shrinking the size of cells, and increasing the content of soluble substances in the cells, so as to maintain the normal life activities of plants^66^. Analysis of the physiological and biochemical indexes of the samples showed that after different times of stress, the soluble sugar content and soluble protein content of Changbai larch transformed with the LoMYB8 gene had a relatively obvious increase compared with CK, while the MDA content, SOD enzyme activity, and POD enzyme activity of the transgenic plants transformed with LoMYB8 showed an upward trend with the prolongation of the stress time.

### Differentially expressed genes under PEG treatment of MYB transgenic plants

**Figure 12:**
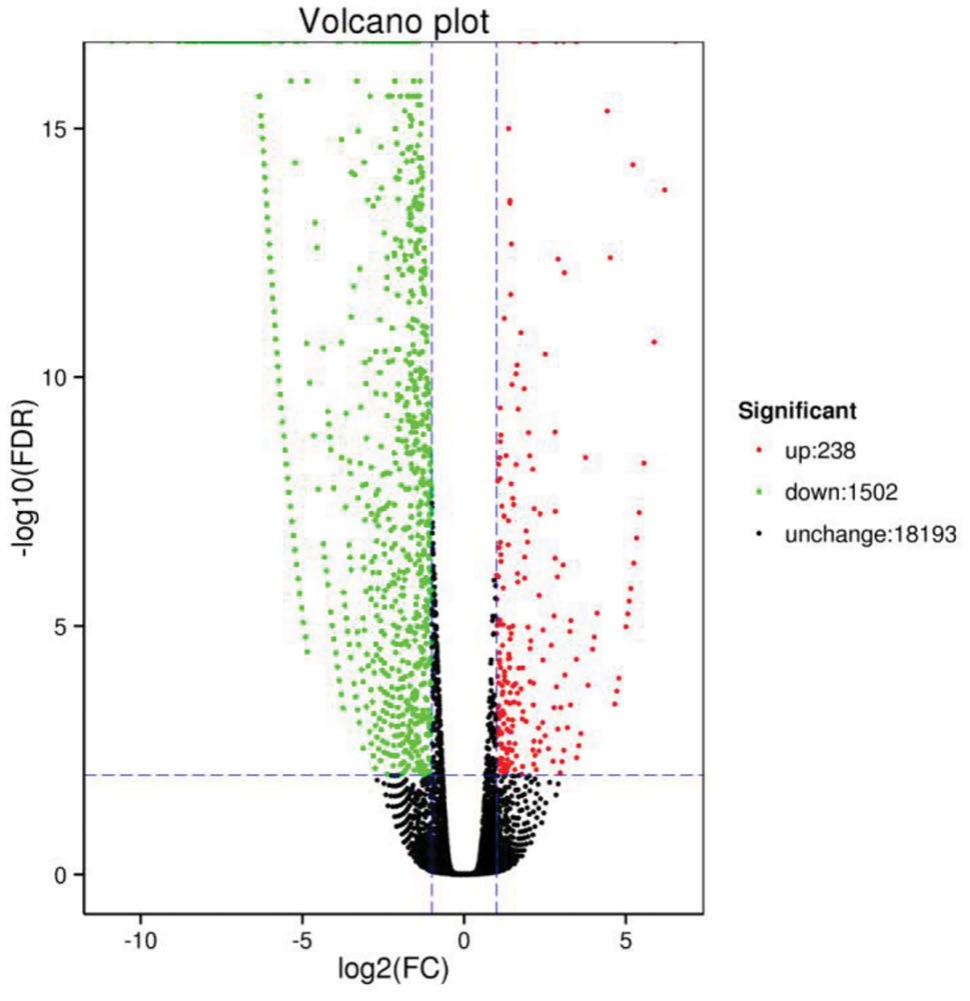
Volcano plot of differentially expressed genes. *Note*: The abscissa in the figure represents the logarithm of the multiple differences in gene expression between the two samples, and the ordinate represents the negative logarithm of the false discovery rate. Each dot in the figure represents a gene. Black represents genes with no significant difference in expression, red represents genes with significant differences in expression, red represents genes with upregulated expression, and green represents genes with downregulated expression.

A total of 1740 differentially expressed genes were found in the transiently transformed plants at 48 h of PEG treatment, of which 238 genes were upregulated and 1502 genes were downregulated compared with those in the control plants. The differentially expressed genes are shown in a volcano plot (Fig. 12). There were more downregulated genes than upregulated genes in the transiently transformed plants.

**Figure 13:**
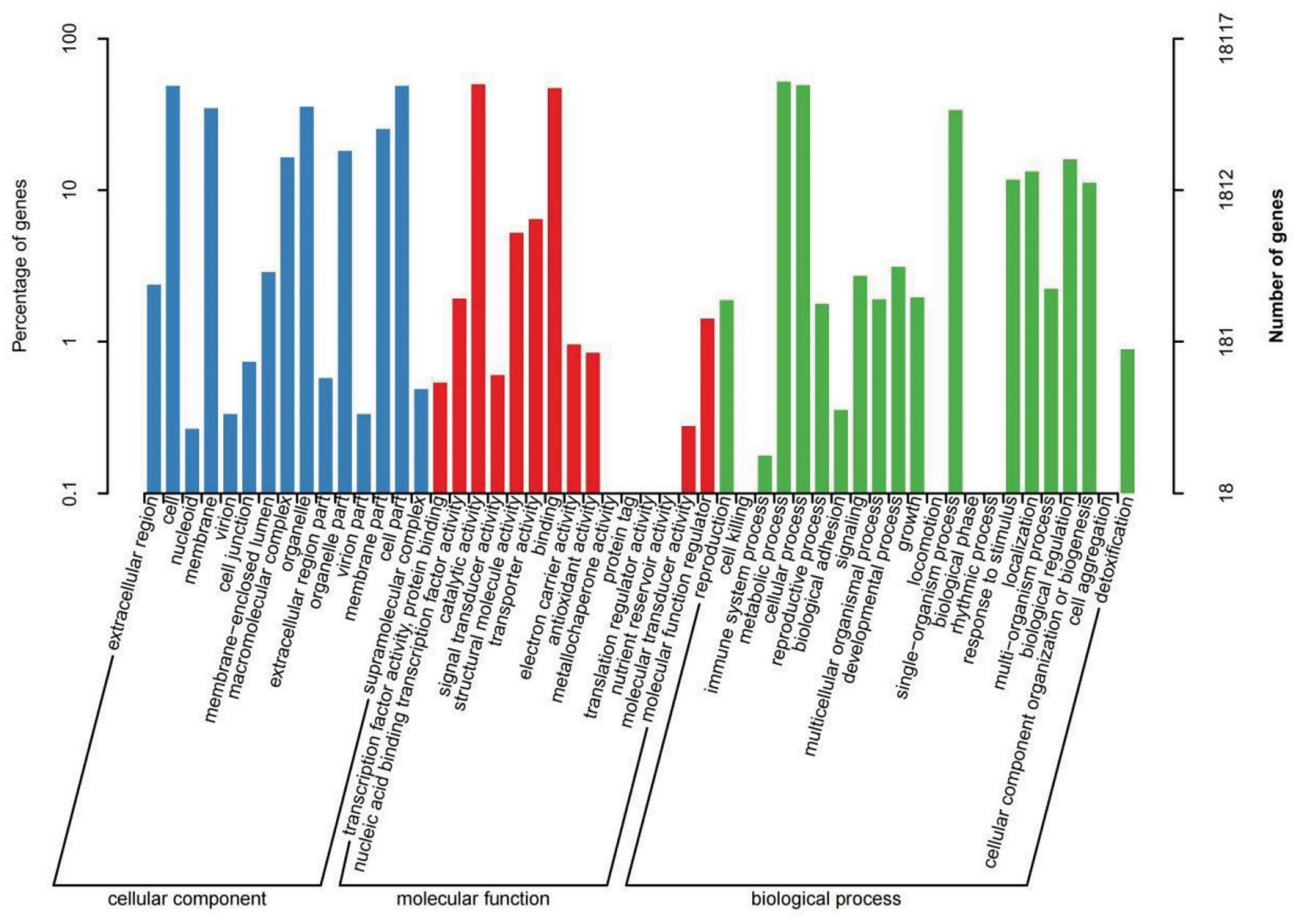
Differentially expressed gene GO analysis results.

As shown in Fig. 13, the GO enrichment classification results of the samples showed that 1227 differentially expressed genes obtained GO annotation by MYB8-T48 compared with CK-T48. The figure shows that MYB8-T48 is enriched with 16, 13 and 17 biological functions in cellular component, molecular function and biological processe, respectively, and 46 biological functions in total. More differential genes were accumulated in cell and cell part, accounting for 64.79% and 64.47% of the total number, respectively. Among the molecular function, the number of differentially expressed genes was 53.30% and 48.41%, respectively. In the biological process, more differentially expressed genes were enriched in metabolic process and cellular process, accounting for 66.01% and 64.30% of the total number, respectively.

Pathway enrichment analysis, which assess the presence of differentially expressed genes in certain pathways (overpresentation), was used to determine the metabolic pathways and signalling pathways in which the differentially expressed genes in the transiently transformed and control plants subjected to PEG-simulated drought stress were involved ^29^. A total of 814 differentially expressed genes were found through the comparison of MYB8–48 h and CK–48 h plants and were annotated using the KEGG database, which indicated that they were involved in 98 metabolic pathways. More than 16 differentially expressed genes were annotated to 23 KEGG pathways (Table 4). A total of 168 differentially expressed genes were annotated to ribosomal pathways, accounting for 20.64% of the total number of differentially expressed genes successfully annotated using KEGG. A total of 38 and 33 differentially expressed genes were annotated to carbon metabolism and amino acid biosynthetic pathways, respectively. The top 23 metabolic pathways to which the differentially expressed genes were annotated were mostly related to the synthesis and metabolism of carbohydrates, amino acids, and flavonoids, and some of them were related to the pathways for the synthesis and metabolism of the substances used in signal transduction, photosynthesis, respiration, and oxidation. In addition, 9 of the pathways showed significant differences with a corrected *P*-value less than 0.05, most of which were related to the metabolism of carbohydrates and amino acids. Therefore, KEGG metabolic pathway enrichment analysis showed that the differences in the differentially expressed genes were mostly related to energy synthesis and metabolism and antioxidant pathways.

**Table 4:** Annotated pathways with more than 16 differentially expressed genes.

Rank	No	Pathways	Amount	Proportion of total KEGG annotated genes (%)	Significant	*P*-value
1	ko03010	Ribosome	168	20.64	Yes	5.39E-10
2	ko01200	Carbon metabolism	91	11.18	Yes	6.82E-09
3	ko01230	Biosynthesis of amino acids	55	6.76		
4	ko00190	Oxidative phosphorylation	54	6.63	Yes	3.10E-05
5	ko00010	Glycolysis/Gluconeogenesis	43	5.28	Yes	0.001386685
6	ko00020	Citrate cycle (TCA cycle)	32	3.93	Yes	0.000859507
7	ko00620	Pyruvate metabolism	31	3.81	Yes	0.019768423
8	ko00940	Phenylpropanoid biosynthesis	28	3.44		
9	ko00500	Starch and sucrose metabolism	25	3.07		
10	ko00630	Glyoxylate and dicarboxylate metabolism	25	3.07		
11	ko04146	Peroxisome	25	3.07		
12	ko04141	Protein processing in endoplasmic reticulum	24	2.95		
13	ko00270	Cysteine and methionine metabolism	23	2.83		
14	ko00710	Carbon fixation in photosynthetic organisms	23	2.83	Yes	0.036712129
15	ko00280	Valine, leucine and isoleucine degradation	23	2.83	Yes	0.031792277
16	ko01210	2-Oxocarboxylic acid metabolism	22	2.70		
17	ko01212	Fatty acid metabolism	21	2.58		
18	ko00640	Propanoate metabolism	19	2.33	Yes	0.002904902
19	ko00071	Fatty acid degradation	18	2.21		
20	ko00030	Pentose phosphate pathway	17	2.09		
21	ko00040	Pentose and glucuronate interconversions	17	2.09		
22	ko00250	Alanine, aspartate and glutamate metabolism	17	2.09		
23	ko04144	Endocytosis	16	1.97		

### Analysis of differential metabolites

**Figure 14:**
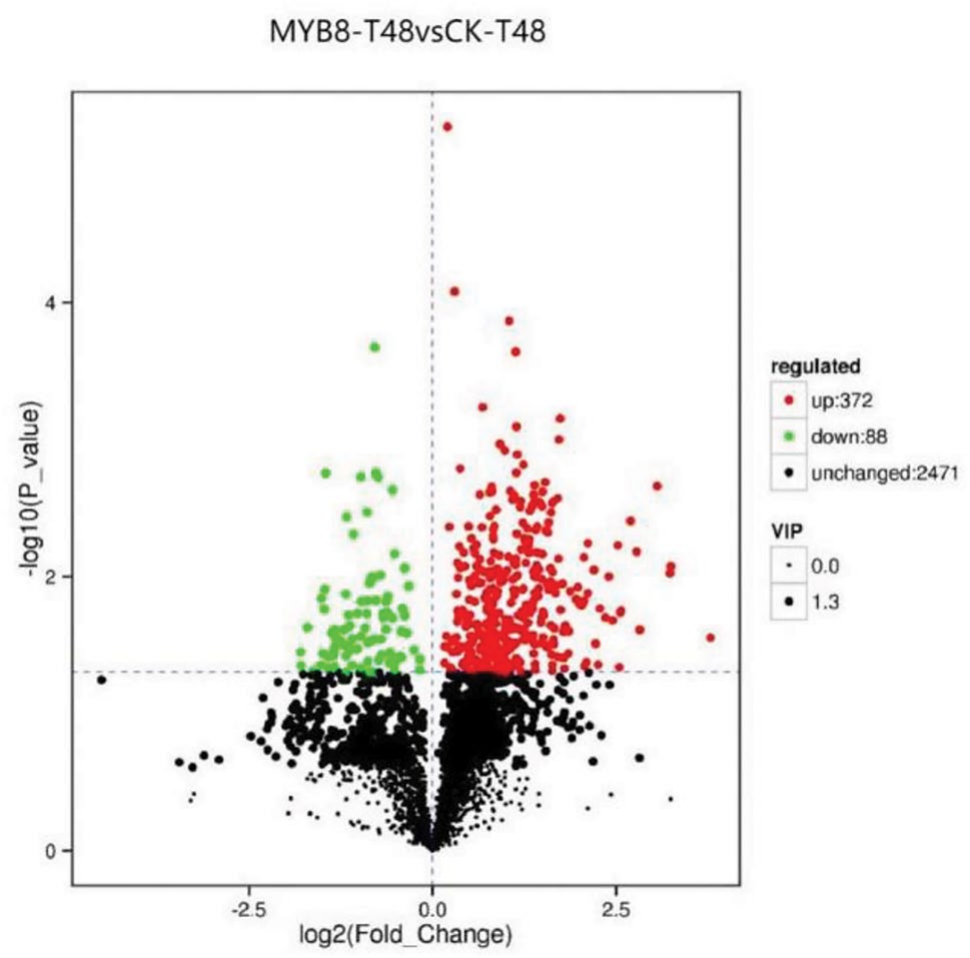
Volcano plot of differential metabolites. *Note*: Each dot in the figure represents a metabolite, red represents the upregulated differential metabolites, green represents the downregulated differential metabolites, and black represents the insignificantly differential metabolites. The abscissa represents the change in the difference multiple of metabolites, and the ordinate represents the logarithm value of *p*-value with base 10. The size of the dot represents the VIP value and the reliability of the metabolite. The larger the VIP value, the more reliable it is.

**Figure 15:**
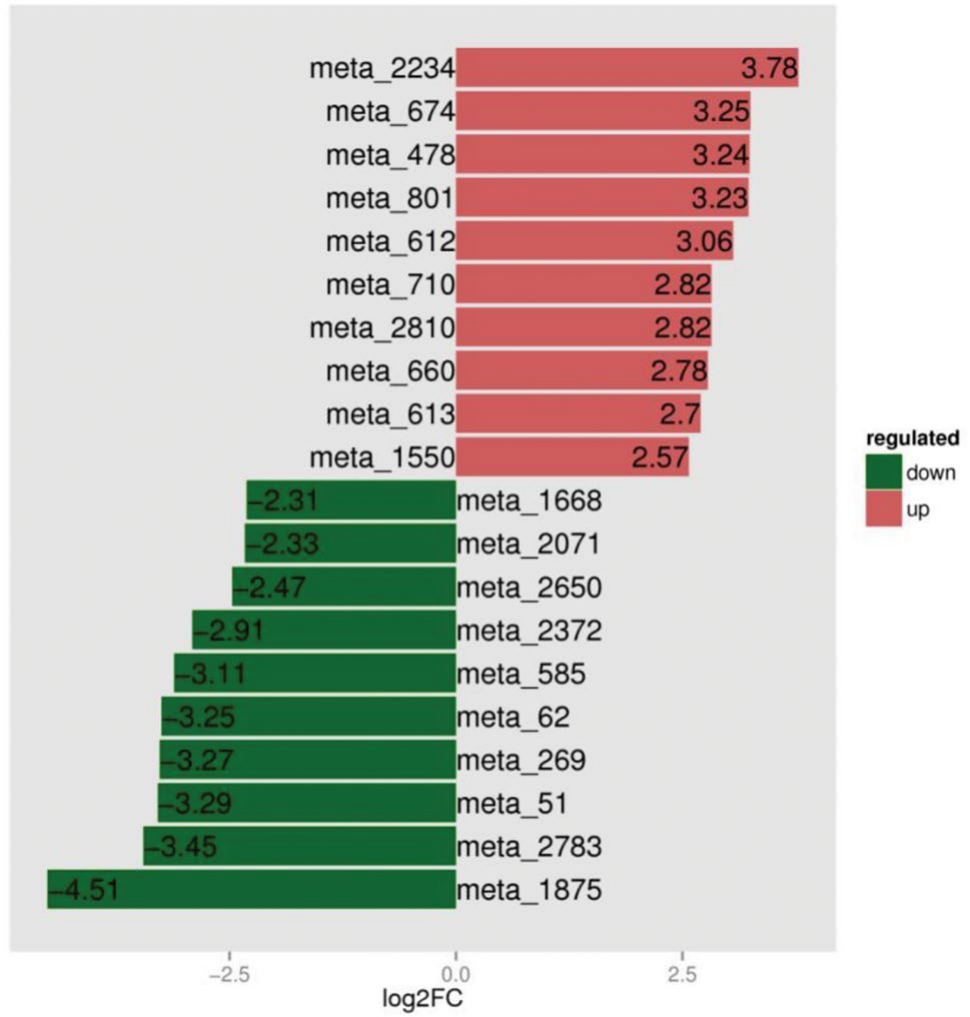
Plot of difference multipliers.

Under PEG-simulated stress, the levels of many metabolites in the transiently transformed and control plants differed. A total of 460 metabolites were differentially regulated between the transiently transformed and control plants (Fig. 14). There were many metabolites upregulated in the transiently transformed plants compared with those in the control plants, and there were many metabolites that showed no significant difference between the two groups. A total of 460 differentially regulated metabolites were detected, of which 80.87% were upregulated. The top 10 upregulated and downregulated metabolites with multiple-fold differences are shown in Fig. 13. Among the top 10 upregulated metabolites (Fig. 15), meta_710 and meta_478 were annotated as benzyl butyl phthalate and amino acids, respectively, and the rest were unknown metabolites. Among the top 10 downregulated metabolites, only meta_51, meta_269, and meta_62 were successfully annotated as mevalonolactone, dacarbazine, and 2-methoxybenzoic acid, respectively.

To explore the function of the differential metabolites we detected in response to drought stress, KEGG pathway enrichment analysis was performed. Among these, 29 differentially regulated metabolites were successfully annotated via KEGG (Table 5), and the metabolites were annotated into 34 metabolic pathways. Over 10% of the annotated metabolites were involved in 12 metabolic pathways, including metabolic pathway, secondary metabolite biosynthesis, glycerophospholipid metabolism, tyrosine metabolism, ABC transporter, phenylpropanoid biosynthesis, carbon metabolism, isoquinoline alkaloid biosynthesis, vitamin B6 metabolism, amino acid biosynthesis, phenylalanine metabolism, and the biosynthesis of phenylalanine, tyrosine, and tryptophan. One differentially regulated metabolite was annotated to each of the following pathways: flavone and flavonoid biosynthesis, pentose phosphate pathway, α-linolenic acid metabolism, arachidonic acid metabolism, and fatty acid biosynthesis.

**Table 5:** Number of KEGG annotations for differential metabolites.

Rank	No	Metabolic pathways	Amount
1	ko01100	Metabolic pathways	15
2	ko01110	Biosynthesis of secondary metabolites	11
3	ko00564	Glycerophospholipid metabolism	5
4	ko00350	Tyrosine metabolism	4
5	ko02010	ABC transporter	3
6	ko00940	Phenylpropanoid biosynthesis	3
7	ko01200	Carbon metabolism	3
8	ko00950	Isoquinoline alkaloid biosynthesis	3
9	ko00750	Vitamin B6 metabolism	3
10	ko01230	Biosynthesis of amino acids	3
11	ko00360	Phenylalanine metabolism	3
12	ko00400	Phenylalanine, tyrosine and tryptophan biosynthesis	3
13	ko00941	Flavonoid biosynthesis	2
14	ko00650	Butanoate metabolism	2
15	ko00030	Pentose phosphate pathway	2
16	ko00970	Aminoacyl-tRNA biosynthesis	2

## Discussion

*LoMYB8* is an R2R3-MYB transcription factor with two R structures. R2R3-MYB transcription factors play an important role in controlling plant growth processes, including primary and secondary metabolism, cell growth and development, and responses to abiotic and biotic stresses^30^. Some MYB genes play a role in the drought response by regulating lateral root growth. In Arabidopsis, the *AtMYB60* and *AtMYB96* genes are involved in the regulation of lateral root growth. Auxin induces the expression of *AtMYB60* in the roots, and the overexpression of this gene in *Arabidopsis* plants growing on MS medium containing mannitol resulted in greater root mass^31^. Huang found a gene, namely, *NbPHAN*, that controls leaf development and drought tolerance in *Nicotiana benthamiana*^32^. NbPHAN belongs to the AS1-RS2-PHAN (ARP) protein complex in the R2R3-type MYB subfamily^30^. The *NbPHAN* gene in the newly emerged young leaves of *N. benthamiana* plants was silenced by means of virus-induced gene silencing (VIGS), which resulted in a change in leaf shape and abnormal growth of the blades along the main veins, while the other organs of the plants remained normal. These plants showed a weakened tolerance to drought stress and increased water loss, but their stomatal density was unchanged. Silencing of the *NbPHAN* gene lowered the expression of stress-related genes that are usually expressed at a high level under water deficit conditions, such as genes involved in polyamine biosynthesis and reactive oxygen detoxification. Additionally, under water deficit conditions, compared with that in nonsilenced plants, the expression level of *NbDREB* but not *NbAREB* decreased in the silenced plants, indicating that *NbPHAN* plays a role in the response to drought stress through an ABA-independent mechanism^32^.

Plant hormones are highly sensitive physiological agents in response to drought stress. In order to ensure normal metabolism, growth, and development, a variety of hormones work coordinately to regulate the physiological responses and gene expression in plants through changes in their concentrations^33,33^. Research has indicated that drought stress regulates the expression of numerous genes in plants, with a significant portion responding to ABA^35^. In this study, we observed that the DEGs in the ABA and auxin signaling pathways were the most prevalent, which aligns with findings from a study that simulated drought stress in potatoes using PEG^36^. Within the ABA pathway, PP2C were down-regulated after drought stress. Research conducted on Populus euphratica has demonstrated that PP2C plays a negative regulatory role in the ABA signaling pathway, and its overexpression reduces plant tolerance^37^, which is consistent with the findings of this study. It is worth noting that the expression of ABA response factor genes showed both upregulated and downregulated, indicating potential differences in the expression patterns of different genes under drought stress. Additionally, an indole-3-acetate amide synthase gene was down-regulated after drought stress, which is consistent with the results of PEG simulation on drought-stressed potatoes^37^. The stability and activity of auxin response factors are regulated by auxin itself. The indole-3-acetate amide synthase gene is an early auxin-responsive gene that plays a crucial role in plant growth and development^38^.

Drought stress usually causes the accumulation of soluble sugars, which play a role in signal transduction and osmotic regulation^39^. The results of our study showed that under PEG-simulated drought stress, the soluble sugar content gradually increased with an increase in stress duration. Under normal growth conditions, the concentration of ROS in plants is very low. Drought stress increases the ROS content in plants, causing oxidative damage^40^. Through the enzymatic protection system, plants use POD to remove active oxygen, thereby protecting the membrane system from damage^41^. Under drought conditions, the permeability of the cell membrane changes, leading to an increase in the relative conductivity and peroxidation of membrane lipids, which results in the production of MDA. The increase in MDA content causes damage to cells^42^. Therefore, the MDA content can be used as an indicator of the degree of damage to plants under drought stress, which indirectly reflects the drought resistance of plants; that is, the higher the MDA content, the greater the damage caused in plants, and the lower the drought resistance^43^.This suggested to a certain extent that LoMYB8 might slow the increase in MDA accumulation in the transiently transformed plants, regulate the response of plants to drought stress, and enhance the short-term drought resistance of plants.

Under drought stress, plants experience an excessive accumulation of ROS, leading to oxidative damage and inhibition of photosynthesis^44^. POD, an impotrant oxidoreductase, plays a regulatory role in plants by catalyzing the redox process and maintaining the balance of H_2_O_2_^45^. Previous studies have shown that enhanced POD activity can enhance plants' resistance to oxidative stress and drought^46^. Similarly, Nikoleta-Kleio demonstrated that applying kaolin clay particles and other substances to young olive trees could elevate POD activity^47^, effectively alleviating water deficiency-induced stress. Furthermore, GST functions as an active oxygen scavenger, and research by George revealed that the PjGSTU1 protein exhibits glutathione transferase activity^48^. Their findings indicated that, PjGSTU1 transgenic tobacco plants exhibited higher survival rates than the control group under drought stress, suggesting its potential role in scavenging reactive oxygen species. In our experiment, the physiological and biochemical indicators in the transiently transformed plants carrying the *LoMYB8* gene and control plants of *Larix *spp. before and after drought stress were determined. The results showed that the soluble sugar content, soluble protein content, MDA content, SOD activity, and POD activity increased in all the plants, which reflects a universal change in plants under drought stress. This conclusion is consistent with that drawn by other researchers. For example, Cui found that the contents of soluble sugar, soluble protein, and MDA in rice and *Arabidopsis* plants under drought stress were higher than those in unstressed control plants^49^. Wang reported that the activity of SOD and POD in maize under drought stress^50^. In our experiment, under drought stress, the soluble sugar content, soluble protein content, SOD activity, and POD activity in the transiently transformed plants overexpressing the *LoMYB8* gene were higher than those in the control plants, while the increase in MDA in the transiently transformed plants was less than that in the control plants, indicating that the transiently transformed *Larix *spp. plants overexpressing the *LoMYB8* gene had stronger drought resistance than the control plants. The changes of physiological and biochemical indexes of transient transformed plants under drought stress in this study were consistent with those in previous studies^51^.

In a study conducted on transiently transformed *Larix *spp. plants overexpressing the *LoMYB8* gene, differentially expressed genes and differentially regulated metabolites were found. These genes and metabolites were annotated to various pathways related to energy synthesis and metabolism, signal transduction, and synthesis and metabolism of flavonoids. These pathways include glycolysis^52^, gluconeogenesis^53^, pyruvate metabolism^54^, pentose phosphate pathway^55^, phenylpropanoid biosynthesis^56^, flavonoid biosynthesis^57^, and flavone and flavonol biosynthesis^58^. These metabolites were shown to vbe inolved in the drought resistance response of plants after drought stress.

### Supplementary Information

Supplementary Information.

## Data Availability

The raw sequence data reported in this paper have been deposited in the Genome Sequence Archive (Genomics, Proteomics & Bioinformatics 2021) in National Genomics Data Center (Nucleic Acids Res 2022), China National Center for Bioinformation / Beijing Institute of Genomics, Chinese Academy of Sciences (GSA: CRA009047) that are publicly accessible at https://ngdc.cncb.ac.cn/gsa.
