# Supplementary material for: Function of MYB8 in larch under PEG simulated drought stress
Source: Sci Rep. 2024 May 17;14:11290. doi: 10.1038/s41598-024-61510-8 (PMC11101485; doi:10.1038/s41598-024-61510-8)
Supplement: Supplementary file 1 — Supplementary Information. [file 41598_2024_61510_MOESM1_ESM.docx]

attachment Table1 Sample Details

| Sample name | Test results | Nanodrop Detection Concentration(ng/ul) | volumetric(ul) | Total (ug) | OD260/280 | OD260/230 | 28S/18S |
| --- | --- | --- | --- | --- | --- | --- | --- |
| CK-1 | A | 473.24 | 30 | 14.2 | 1.98 | 1.38 | 2.66 |
| CK-2 | A | 404.93 | 30 | 12.2 | 1.78 | 0.96 | 2.64 |
| CK-3 | A | 350.75 | 30 | 10.5 | 1.77 | 0.66 | 2.08 |
| T1-1 | A | 368.37 | 30 | 11.1 | 2.02 | 1.39 | 2.96 |
| T1-2 | A | 446.68 | 30 | 13.4 | 2.03 | 1.37 | 2.70 |
| T1-3 | A | 451.81 | 30 | 13.6 | 2.10 | 1.74 | 2.45 |
| T2-1 | A | 465.91 | 30 | 14.1 | 2.06 | 1.39 | 2.77 |
| T2-2 | A | 571.82 | 30 | 17.2 | 1.99 | 1.38 | 2.68 |
| T2-3 | A | 357.36 | 30 | 10.7 | 1.96 | 1.11 | 2.91 |
| T3-1 | A | 525.11 | 30 | 15.8 | 2.09 | 2.02 | 2.36 |
| T3-2 | A | 466.71 | 30 | 14.0 | 1.95 | 1.32 | 2.41 |
| T3-3 | A | 448.61 | 30 | 13.5 | 1.98 | 1.82 | 2.40 |

attachment Table 2 CSS data statistics table

| Samples | cDNA size | CCS Number | Read Bases of CCS | Mean Read Length of CCS | Mean Number of Passes |
| --- | --- | --- | --- | --- | --- |
| T | 1-6K | 786,492 | 2,603,443,180 | 3,310 | 36 |
| T | ALL | 786,492 | 2,603,443,180 | 3,310 | 36 |
